# Bovine Tooth Discoloration Induced by Endodontic Filling Materials for Primary Teeth

**DOI:** 10.1155/2017/7401962

**Published:** 2017-04-05

**Authors:** Samantha Rodrigues Xavier, Katerine Jahnecke Pilownic, Andressa Heberle Gastmann, Mariana Silveira Echeverria, Ana Regina Romano, Fernanda Geraldo Pappen

**Affiliations:** Graduate Program in Dentistry, Federal University of Pelotas, Pelotas, RS, Brazil

## Abstract

*Objective.* This study evaluated the discoloration potential of endodontic materials used in primary teeth.* Material and Methods.* Dentine-enamel blocks were prepared from 75 bovine teeth, assorted in five experimental groups (*n* = 15). The tested materials included an MTA-based material; zinc oxide and eugenol cement (ZOE); Vitapex; and calcium hydroxide thickened with zinc oxide (Calen + ZO). The color measurements were performed using a spectrophotometer at the following intervals: prior to (T0) and after placement of the filling (T1) and after 1 week (T2), 1 month (T3), 3 months (T4), 6 months (T5), and 9 months (T6). Data were submitted to ANOVA with repeated measures and Tukey's test.* Results.* The time had a significant effect on the color variation (Δ*E*_00_^⁎^) (*p* < 0.0001). The effect of the materials on the color variation (Δ*E*_00_^⁎^) was statistically significant (*p* = 0.004). Interactions between time and materials demonstrated a significant effect on the values (Δ*E*_00_^⁎^) (*p* < 0.0001). The ZOE cement showed the highest darkening effect (*p* = 0.018).* Conclusion.* The MTA-based material showed the smallest discoloration during the experimental time; however, it was similar to the other materials and to the control group. Zinc oxide and eugenol showed higher discoloration.

## 1. Introduction

Endodontic therapy should not focus solely on biological and functional aspects; aesthetic considerations must also be seen in primary teeth [1, 2]. Children are conscious about their dental aesthetic appearance and that of others [3]. Dental discoloration is the main negative perception of children in relation to their mouth [4]. The main causes of intrinsic tooth discoloration related to endodontic treatment are decomposition of the necrotic pulp tissue, hemorrhage into the pulp chamber, endodontic medications, and filling materials [5, 6].

Crown discoloration related to endodontic filling materials [6, 7] is associated with the material time to contact the tooth structure, as well as the potential chromogenic materials used in the treatment [8]. The materials commonly used in Endodontics for primary teeth are zinc oxide eugenol (ZOE), iodoform pastes, and calcium hydroxide pastes [9, 10]. Recently, a ready-to-use MTA-based (Mineral Trioxide Aggregate) root filling material for primary teeth has been developed (Angelus®, Londrina, PR, Brazil). Unpublished data have shown satisfactory cytotoxicity, radiopacity, pH, and antimicrobial capacity.

The aim of the present study was to investigate the discoloration potential of some endodontic filling materials for primary teeth using bovine tooth model. The tested hypotheses were that there would be no difference in discoloration among the tested materials after 9 months and that all materials would show a similar progression of discoloration over time.

## 2. Materials and Methods

### 2.1. Specimen Preparation

The present study followed the method described by Lenherr et al. [1]. Seventy-five bovine incisors were extracted, cleaned, and stored in water at room temperature. Next, the roots was removed and the labial surface of each tooth was cleaned with scalers. A cuboid enamel-dentine block (10 × 10 mm) was prepared from the middle third of each crown using a bur 4138 (KG Sorensen, São Paulo, SP, Brazil).

**Figure 1:**
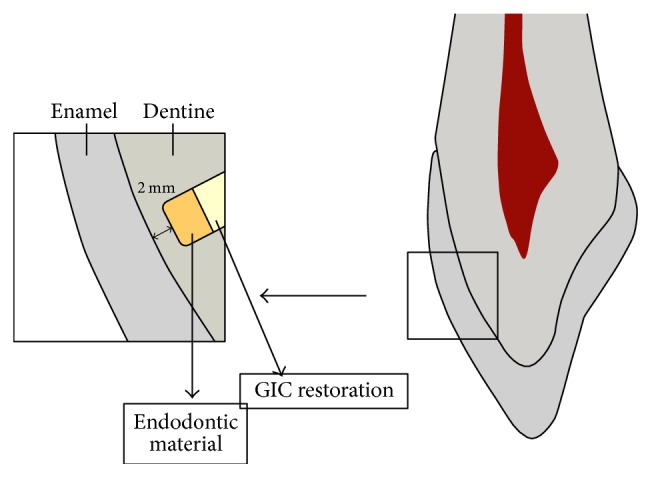
Schematic presentation of performed procedures prior to color measurements.

The height of each block was standardized at 3.5 ± 0.1 mm, measured with an endodontic scale. A cylindrical-shaped hole with a diameter of 4 mm was drilled with a bur 1014 (KG Sorensen) in the center of each specimen to leave 2 mm of the labial tooth structure (Figure 1). The specimens were immersed in 1% sodium hypochlorite (Asfer, São Caetano do Sul, SP, Brazil) for 30 minutes followed by 17% EDTA (Biodinâmica, Ibiporã, PR, Brazil) for 3 minutes to remove the smear layer. After a final three minutes in sodium hypochlorite, the specimens were stored in sterilized distilled water.

The tested materials included a MTA-based material (Angelus, Londrina, PR, Brazil), zinc oxide and eugenol cement (ZOE) (Biodinâmica Química e Farmacêutica Ltda., Ibiporã, PR, Brazil), a premixed calcium hydroxide and iodoform paste (Vitapex, Neo Dental International Inc., Federal Way, OH, USA), and 1.0 g of a premixed calcium hydroxide and polyethylene glycol-based paste (Calen, S.S. White, Rio de Janeiro, RJ, Brazil) thickened with 1.0 g zinc oxide (Biodinâmica Química e Farmacêutica Ltda., Ibiporã, PR, Brazil) (Calen + ZO).

The specimens were randomly divided into 5 groups (*n* = 15), and the endodontic filling materials were placed into the cavities (Table 1). All cavities, including those in the control group, were sealed with glass ionomer cement (Vitro Fill LC, DFL, Rio de Janeiro, RJ, Brazil). The polymerization was carried out with an LED light curing (800 mW/c^2^ irradiance) (Emitter, Schuster Ltda., Santa Maria, RS, Brazil) for 20 s.

Every specimen was placed into a single 15 mL Falcon tube (Kasvi, Curitiba, PR, Brazil) with water. The tubes were stored at 37°C, in the dark, for the following period up to 9 months.

### 2.2. Color Determination

In a dark room, color measurements were recorded using a spectrophotometer (Vita Easyshade, Vita-Zahnfabrik, Oberding, BV, Germany), under standardized conditions, using a measuring station, which permits that all spectrophotometer readings are done in the same tooth area. The instrument was calibrated before the measurement in each group.

Seven sessions of color measurements were performed at the following intervals: prior to (T0 = baseline) and after (T1) placement of the endodontic filling material and after 1 week (T2), 1 month (T3), 3 months (T4), 6 months (T5), and 9 months (T6). In order to avoid optical changes caused by dehydration, the excess water was removed briefly by air-drying for 1 s before each measurement. The CIE *L*^*∗*^*a*^*∗*^*b*^*∗*^ data were collected and further analyzed. Color change (Δ*E*_00_^*∗*^) values were calculated using the following formula CIEDE2000 for each specimen [11, 12]:

(1) $$\begin{array}{c} \Delta E_{00}\left(\;L_{1}^{*},a_{1}^{*},b_{1}^{*};L_{2}^{*},a_{2}^{*},b_{2}^{*}\;\right)=\Delta E_{00}^{12}=\Delta E_{00}. \end{array}$$

The *L*^*∗*^ values describe the luminosity, which varies from black (0) to white (100), while the *a*^*∗*^ and *b*^*∗*^ values indicate the chromatic direction red/green and blue/yellow, respectively [11, 12]. The smaller the Δ*E*_00_ value, the lower the color difference between the initial color and the final color of the tooth over time.

### 2.3. Statistical Analysis

Considering the statistical analysis, data that violated the assumptions of equality of variances and normal distribution of errors were ranked and analyzed by repeated measures ANOVA. Additionally, the mean values of all groups were compared using the Tukey multiple comparison test to evaluate the effect of the factors of time and material on the dental discoloration (*p* = 0.05) by using SPSS 16.0 software (SPSS Inc., Chicago, IL).

## 3. Results

Table 2 shows the results for color variation (Δ*E*_00_^*∗*^) with respect to the effects of time and their interaction with the experimental groups. The time had a significant effect on color change values (Δ*E*_00_^*∗*^) (*p* < 0.0001). The effect of the materials on color change (Δ*E*_00_^*∗*^) was also statistically significant (*p* = 0.004). The interactions between time and materials demonstrated a significant effect on Δ*E*_00_^*∗*^ values (*p* < 0.0001). The average color change at different times for all experimental groups is described in Table 3.

The MTA-based material showed the smallest Δ*E*_00_^*∗*^ values during the experimental time; however, it was similar to the other materials and to the control group (*p* > 0.05). Zinc oxide and eugenol showed higher discoloration (*p* = 0.018).

## 4. Discussion

In the present study, all materials caused color changes, even in the first experimental time. The evaluated endodontic filling materials provided Δ*E*_00_^*∗*^ values higher than 1.8, which is the clinical threshold for acceptability of color differences [12]. All materials except the ZOE cement had similar mean discoloration to the control group. Children are able to realize aesthetic changes, and tooth discoloration is the main negative perception [3, 4]. For this reason, it is important to assess the staining ability of endodontic filling materials for primary teeth when there are no studies available in the literature [10], since endodontic filling materials may induce tooth discoloration [6, 7], particularly if applied in the pulp chamber and above the gingival margin.

A bovine tooth model was used to avoid variability in human tooth morphology [1]. Due to the number of dentinal tubules, bovine teeth have no significant difference compared to human teeth [13]. For the assessment of discoloration, enamel-dentine blocks were used in order to standardize the thickness of the dentine in contact with the material, as well as the size of the cavity and the amount of material employed [1, 14].

Color determination can be performed through visual techniques or the use of instruments such as a spectrophotometer or a colorimeter. Despite visual assessment being the most used in clinical practice, it is based on subjective measurements using a visual color scale to compare shades [15]. The ideal method for color measurement should be reliable, reproducible, and easy to perform in order to allow comparison of the color measurements at different times [16]. In the present study, the Vita Easyshade Device was chosen due to its high data stability and excellent repeatability [1, 17].

There are two main limitations to evaluating color differences: perceptibility threshold and acceptability threshold [18]. The use of a suitable color difference formula is important for obtaining a correct correlation of awareness and acceptance by the color difference values obtained by a spectrophotometer. Thus, the use of the CIEDE2000 system, recommended by CIE using the color difference formula (*E*_00_) [19], is considered more appropriate than the CIE *L*^*∗*^*a*^*∗*^*b*^*∗*^ formula [20]. In the CIEDE2000 formula, specific corrections are included for the nonuniformity of the CIE *L*^*∗*^*a*^*∗*^*b*^*∗*^ space (weighing functions SL, SC, and SH)) and parameters that account for the influence of illuminating and viewing conditions in the evaluation of color difference (parametric factors KL, KC, and KH) [21].

The results of the present study showed that even in the control group the color difference could be perceived by the human eye. This result occurred because the baseline for the color variation measurements was the bovine teeth, in the moment that the cavities were not restored. Consequently, the color alteration was possibly influenced by the postendodontic treatment restoration [22, 23]. Among the tested materials, the ZOE cement showed the highest color change over time, reaching Δ*E*_00_^*∗*^ = 9.59 after 270 days. This result is similar to previous studies [6, 7] and probably occurred due to the chromogenic potential of the ZOE cement, attributed to the unstable chemical bond between ZnO and eugenol. Even after the end of the setting reaction, eugenol release leads to self-oxidation and becomes darker with time [6, 7, 24].

Calcium hydroxide has its discoloration capacity changed according to the components added to its formula [1]. Previous studies have demonstrated that pure calcium hydroxide caused no visible discoloration in any experimental time [1, 25]. In the present study, the Vitapex, composed of calcium hydroxide and iodoform, and calcium hydroxide paste thickened with zinc oxide showed Δ*E*_00_^*∗*^ values exceeding the perceptibility threshold [14]. This color change caused by Calen + ZO may be attributed to the zinc oxide present in its formula, while, in the Vitapex group, it may be related to the production of a yellowish-brown discoloration generated by iodoform, compromising the aesthetics [26].

Other studies have shown that conventional MTA, both white and grey MTA formulas, which has bismuth oxide as an opacifier, are able to induce dental discoloration [1, 24]. This is due to crystals of bismuth atoms under light conditions [27]. The MTA-based material had tungstate as an opacifier, and previous studies with Portland cement with 20% calcium tungstate showed no discoloration over time [28]. Maybe this is the reason for the values found over time with the MTA-based material (Δ*E*_00_^*∗*^ = 3.91), which were lower compared to the ZOE (Δ*E*_00_^*∗*^ = 9.59). Therefore, in terms of aesthetics, the use of this material appears to be favorable.

Up to now, there is limited information about the properties of root canal filling materials used in primary teeth. Additional in vitro, ex vivo, and in vivo researches must be conducted to evaluate the performance of the MTA-based material in order to confirm its use in endodontic therapy.

## 5. Conclusions

The MTA-based material showed the smallest discoloration during the experimental time; however, it was similar to the other materials and to the control group. Zinc oxide and eugenol showed higher discoloration.

## Figures and Tables

**Table 1:** Distribution of experimental materials.

Group	N	Material	Manufacturer
I	15	MTA-based material	Angelus, Londrina, PR, Brazil
II	15	Vitapex	Neo Dental International Inc., Federal Way, United States
III	15	Calen thickened with zinc oxide (Calen + ZO)	S.S. White, Artigos Dentários, Rio de Janeiro, RJ, Brazil
IV	15	Zinc oxide eugenol cement	Biodinâmica, Ibiporã, PR, Brazil
Control	15	No endodontic material	—

**Table 2:** Color variation results (Δ*E*_00_^*∗*^) by ANOVA with repeated measures with regard to the effects of weather and their interaction groups.

Variation	Type III sum squares	Df	Mean square	*F*	(*p* value)
Intercept	7783.437	1	7783.437	295.189	0.000
Material	441.254	4	110.313	4.184	0.004
Error	1845.734	70	26.368		
Time	300.345	5	60.069	8.765	0.000
Time *∗* material	938.643	20	46.932	6.848	0.000
Error	2398.756	350	6.854		

**Table 3:** Mean and standard deviation of variation in color values (Δ*E*_00_^*∗*^ in the different experimental groups.

Group	Material	After restoration	1 week	1 month	3 months	6 months	9 months
I	MTA-based material	2.71 ± 1.64^a^	2.48 ± 1.29^a^	2.68 ± 1.09^a^	2.89 ± 1.43^a^	3.47 ± 2.62^a^	3.91 ± 1.89^a^
II	Vitapex	3.23 ± 2.16^a^	2.57 ± 1.07^a^	3.87 ± 1.81^a^	3.66 ± 1.93^ab^	4.56 ± 1.89^a^	3.60 ± 2.48^a^
III	Calen + ZO	3.82 ± 2.25^a^	3.60 ± 3.12^a^	4.63 ± 4.23^a^	6.34 ± 6.60^b^	3.34 ± 2.56^a^	3.19 ± 2.52^a^
IV	ZOE	3.38 ± 1.48^a^	3.58 ± 1.50^a^	3.38 ± 1.56^a^	3.91 ± 1.72^ab^	9.92 ± 6.98^b^	11.63 ± 9.59^b^
Control	Control	3.81 ± 2.73^a^	3.73 ± 1.31^a^	4.11 ± 1.54^a^	3.88 ± 1.36^ab^	4.89 ± 2.65^a^	3.81 ± 1.34^a^

Same letters within the same period of time (column) indicate statistically similar results for the materials (*p* > 0.05).
